# Mapping Antibody Epitopes of the Avian H5N1 Influenza Virus

**DOI:** 10.1371/journal.pmed.1000064

**Published:** 2009-04-21

**Authors:** Hui-Ling Yen, J. S. Malik Peiris

**Affiliations:** 1 Department of Microbiology, The University of Hong Kong, University Pathology Building, Queen Mary Hospital, Hong Kong, Special Administrative Region, People's Republic of China; 2 HKU-Pasteur Research Centre, Hong Kong, Special Administrative Region, People's Republic of China

## Abstract

Hui-Ling Yen and J. S. Malik Peiris discuss a study in *PLoS Medicine* that provides new information on the human antibody repertoire generated in response to H5N1 influenza virus infection.

**Linked Research Article**

This Perspective discusses the following new study published in *PLoS Medicine*:

Khurana S, Suguitan AL Jr., Rivera Y, Simmons CP, Lanzavecchia A, et al. (2009) Antigenic fingerprinting of H5N1 avian influenza using convalescent sera and monoclonal antibodies reveals potential vaccine and diagnostic targets. PLoS Med 6(4): e1000049. doi:10.1371/journal.pmed.1000049

Using whole-genome-fragment phage display libraries, Hana Golding and colleagues identify the viral epitopes recognized by serum antibodies in humans who have recovered from infection with H5N1 avian influenza.

**Glossary**

**Random peptide phage display library**: A technique that can be used to select peptide ligands binding to a target molecule (peptide, protein [e.g., antibody], DNA, or RNA). A library of bacteriophages each expressing a random peptide (e.g., 12 mers) fused to the bacteriophage surface proteins is generated. Bacteriophages that specifically bind to the target molecule are purified through repeated cycles of binding and elution, and the inserts are PCR amplified and sequenced to deduce the peptide that binds to the target molecule. In the paper by Golding et al., this strategy was used to identify the viral epitopes (peptide sequences) recognized by two monoclonal antibodies targeting the H5N1 HA. This method provides fine mapping of antibody epitope to short peptide sequence as compared to the whole-genome-fragment phage display libraries (see below).

**Whole-genome-fragment phage display libraries**: Instead of using random peptides, the cDNA corresponding to the whole genome of a 2004 human H5N1 influenza isolate was used to construct the phage display library. In the study by Golding et al., cDNA of size ranges of 50–200 or 200–1,000 bp was used to construct the phage display library. Convalescent sera from patients with H5N1 disease were used to probe this phage display library, and the virus epitopes binding specific antibodies were identified.

**Clade 1 H5N1 influenza virus**: Phylogenetic analysis of the HA gene of highly pathogenic avian influenza H5N1 viruses has led to the subdivision of these viruses into ten virus clades. Phylogenetic trees are like family trees, and a clade of viruses are a group of viruses that are more closely related genetically. The different genetic clades of virus are generally, but not invariably, antigenically distinct, and these differences are relevant in designing vaccines and in assessing vaccine cross-protection.

Antibodies are a major component of specific immune protection against influenza and remain the established immune correlate of protection for influenza vaccines. The importance of humoral immunity against influenza infection is further highlighted by the apparent success of passive immunotherapy with convalescent sera during the 1918 Spanish influenza pandemic, and more recently by anecdotal reports of treating H5N1 human infection with convalescent sera [1], [2]. Human monoclonal antibodies to H5N1 viruses have been generated from immortalized human memory B cells obtained from patients who recovered from H5N1 disease [3] or with combinational antibody library technologies [4]. Some of these antibodies have broad H5N1 cross-clade reactivity [3], [4] or cross-subtype reactivity to H1 viruses [4], and are effective in suppressing H5N1 virus disease in experimentally infected animals when administered prophylactically or therapeutically [3].

Influenza hemagglutinin (HA), with 16 antigenically distinct subtypes, and neuraminidase (NA), with nine antigenically distinct subtypes, are the major surface glycoproteins targeted by host antibody response. Antibodies against HA may neutralize the virus through blocking viral attachment to the sialyl receptors on host cells or through interfering with HA conformational changes at low pH within the endosome, thereby preventing fusion and uncoating of the virus [5]–[8]. Although anti-NA antibodies cannot provide a sterilizing effect in vivo, they have been shown to reduce viral titers, morbidity, and viral shedding [9]–[12]. M2 is a conserved viral protein abundantly expressed on the infected cell surface, and anti-M2 antibodies may provide broad cross-protection to influenza viruses of different subtypes (known as heterosubtypic immunity) [13].

Although influenza control relies on eliciting protective humoral immunity through vaccination, there is insufficient information on the antibody epitopes on influenza viruses. Much of the available information pertains to antibodies generated from mice rather than humans [14]. Antibody epitopes have been identified from only five of the 11 viral proteins, and most of these epitopes are on the viral HA [14]. Epitope mapping using monoclonal antibodies and the availability of the 3-dimensional structure have identified five antigenic sites in the HA of H3 subtype [15], [16]. Corresponding antigenic sites have also subsequently been mapped to H1 and H2 subtypes [17], [18]. The antibody binding epitopes of the H5 HA epitopes have been mapped using virus escape mutants (viral variants that can escape recognition by the monoclonal antibodies) and are located exclusively in areas corresponding to antigenic sites A and B of H3 HA and the antigenic site Sa of H1 HA [19], [20], at the upper surface of the HA molecule. Furthermore, differences between a low-pathogenic strain (A/Mallard/Pennsylvania/10218/84 [H5N2]) and a recent high-pathogenic strain (A/Vietnam/1203/04 [H5N1]) have been observed, suggesting the potential differences in HA conformations even within the same subtype [19], [20].

## A New Study on Human Antibodies Generated in Response to H5N1

In the current issue of *PLoS Medicine*, Hana Golding and coauthors [21] use whole-genome-fragment phage display libraries (see Glossary) expressing fragments of a clade 1 H5N1 influenza virus (A/Vietnam/1203/04) and a random peptide phage display library to define the conformation-dependent epitopes of two neutralizing human monoclonal antibodies, one with reactivity restricted to clade 1 viruses and the other with capacity for broader cross-clade protection [3]. They go on to define the H5N1 virus reactive antibody epitopes recognized in the convalescent sera from five patients with H5N1 disease collected between 54 and 182 days after hospitalization. H5N1-specific epitopes were identified in HA and NA surface glycoproteins as well as M2e, PB1-F2, and others. To differentiate potential cross-reactive antibody response elicited by previous exposure to H1N1 or H3N2 influenza viruses, control sera obtained from Vietnamese (*n* = 20) and US (*n* = 10) residents with no known exposure to H5N1 virus were also analyzed against the H5N1 whole-genome-fragment phage display library. Cross-reactive epitopes were identified in several H5N1 viral proteins, with strong reactions to peptides in HA and M1 and PA. This study provides much-needed information on the human antibody repertoire generated in response to H5N1 influenza virus infection, and these findings open up new avenues of research.

## New Avenues of Research Arising from This Study

Further work is required to define which of these antibody epitopes elicit antibodies that protect against H5N1 infection, whether such protection spans many of the H5N1 clades and subclades, and whether some of these antibodies provide protection that might even extend to other influenza virus subtypes. It is expected that some of the epitopes in the HA are targets for the neutralizing antibodies; however, it is also important to evaluate whether the antibodies targeting epitopes in NA and M2e may facilitate clearance of H5N1 infection. The protective roles for cross-reactive antibodies targeting NA have been suggested previously [22]. As the NA epitope identified by Golding and colleagues is located in proximity to the enzyme active site, it is possible that the interacting antibody can block NA enzymatic activity and thereby block viral release. Antibodies targeting M2e have been shown to be effective in animal models [23], and an M2e vaccine is currently being evaluated in clinical trials as an universal vaccine for influenza because of presumed broad subtype cross-reactivity induced by such antigens. M2e has previously been reported as being weakly immunogenic, and such antibodies detected after natural influenza infection are of low titer and of short duration [24]. Golding and colleagues found that four H5N1 convalescent sera (collected 54–113 days post-admission) showed strong M2e antibody titers (≥2,500), while the fifth H5N1 convalescent serum collected at 182 days post-admission showed a low antibody titer (of 100). Whether this reflects the short duration of an M2e antibody response needs to be established. Surprisingly, sera from controls with no exposure to H5N1 virus but with high antibody titres to seasonal influenza viruses had no reactivity to these H5N1 M2e epitopes.

It would be important to establish whether these H5N1 M2e epitopes confer protection against other influenza subtypes, including seasonal human influenza viruses. Human H5N1 disease differs from seasonal influenza in disease pathogenesis and in the extent of acute lung injury. Thus we need to understand whether the differences observed between H5N1 convalescent sera and control sera from persons with high titers to seasonal influenza virus are the result of differences in such disease pathology.

Some of the epitopes found to be reactive with convalescent sera from H5N1 patients were also found to cross-react with the control sera from individuals with exposure to seasonal influenza (such as peptide HA-2376-2659). It is important to explore these epitopes further to determine if any of them are conserved across different influenza virus subtypes. It is interesting to note that others have recently derived human monoclonal antibodies that neutralize many (though not all) influenza virus subtypes, and some of these antibodies provide protection in experimentally infected mice [25], [26]. These monoclonal antibodies appear to target conserved domains in the HA1/HA2 stem region and lead to virus neutralization by inhibiting membrane fusion [26], [27]. Studies are needed to further elucidate if some of the cross-reactive HA epitopes found in Golding and colleagues' study are also related to this region. Such information is important in generating passive antibodies and vaccines with the capacity to protect against multiple influenza virus subtypes. These new insights provide a better understanding of antibody epitopes of influenza and are crucial to our efforts to be better prepared for the next pandemic.

## Abbreviations

HA: hemagglutinin
NA: neuraminidase
